# Gut microbiota metabolites, secretory immunoglobulin A and Bayley-III cognitive scores in children from the CHILD Cohort Study

**DOI:** 10.1016/j.bbih.2025.100946

**Published:** 2025-01-15

**Authors:** Aline Davias, Myah Verghese, Sarah L. Bridgman, Hein M. Tun, Catherine J. Field, Matthew Hicks, Jacqueline Pei, Anne Hicks, Theo J. Moraes, Elinor Simons, Stuart E. Turvey, Padmaja Subbarao, James A. Scott, Piushkumar J. Mandhane, Anita L. Kozyrskyj

**Affiliations:** a Edmonton Clinic Health Academy, Department of Pediatrics, Faculty of Medicine and Dentistry, University of Alberta, Edmonton, Canada; b Environmental Epidemiology Applied to Development and Respiratory Health Team, Institute for Advanced Biosciences, University Grenoble Alpes, Inserm, CNRS, 38700, La Tronche, France; c The Jockey Club School of Public Health and Primary Care, Li Ka Shing Institute of Health Sciences, Faculty of Medicine, The Chinese University of Hong Kong, Hong Kong, SAR, China; d Microbiota I-Center (MagIC), Hong Kong, SAR, China; e Department of Agricultural, Food and Nutritional Science, Faculty of Agricultural, Life and Environmental Sciences, University of Alberta, Edmonton, Canada; f Department of Educational Psychology, Faculty of Education, University of Alberta, Edmonton, Canada; g Hospital for Sick Children (SickKids), Department of Pediatrics, University of Toronto, Toronto, Canada; h Children's Hospital Research Institute of Manitoba, Department of Pediatrics and Child Health, University of Manitoba, Winnipeg, Canada; i BC Children's Hospital, Department of Pediatrics, Faculty of Medicine, University of British Columbia, Vancouver, Canada; j Dalla Lana School of Public Health, Division of Occupational and Environmental Health, University of Toronto, Toronto, Canada; k Faculty of Medicine and Health Sciences, UCSI University, Kuala Lumpur, Malaysia

**Keywords:** Bayley-III scale of infant development, Infant gut microbiota, Secretory immunoglobulin A

## Abstract

**Background:**

Dysbiosis of the gut microbiota has been demonstrated in neurodevelopmental disorders but the underlying mechanisms that may explain these associations are poorly understood. Gut secretory immunoglobulin A (SIgA) binds pathogenic microbes, preventing mucosal penetration. Gut microbes also influence SIgA production and its binding characteristics through short-chain fatty acid (SCFA) metabolites, allowing them to regulate the immune response. Serum IgA deficiency has been noted in children with autism spectrum disorders (ASD). In this study, we aimed to determine whether SIgA level in infancy is associated with gut microbiota taxonomy and metabolites, and neurodevelopmental outcomes in preschool children.

**Methods:**

For a subsample of 178 children from the Canadian CHILD Cohort Study, gut microbiota of fecal samples collected at 3–4 months and 12 months was profiled using 16S rRNA sequencing. Gut bacterial metabolites levels and SIgA level were measured by nuclear magnetic resonance (NMR) based metabolomics and SIgA enzyme-linked immunosorbent assay at 3–4 months, respectively. Bayley-III Scale of Infant Development was assessed at 12 and 24 months. We evaluated direct relationships in multiple linear regression models and putative causal relationships in statistical mediation models.

**Results:**

Propionate and butyrate levels at 3–4 months were associated with decreased Bayley cognitive score at 24 months (p-values: 0.01 and 0.02, respectively) in adjusted multiple linear regression models, but when we investigated an indirect relationship mediated by decreased SIgA level at 3–4 months, it did not reach statistical significance (p-values: 0.18 and 0.20, respectively). Lactate level at 3–4 months was associated with increased Bayley cognitive score at 24 months in adjusted multiple linear regression models (p-value: 0.01), but the statistical model mediated by increased SIgA level at 3–4 months did not reach statistical significance neither (p-value: 0.20).

**Conclusions:**

Our study contributes to growing evidence that neurodevelopment is influenced by the infant gut microbiota and that it might involve SIgA level, but larger studies are required.

## Highlights

• Cognitive scores were greater when Proteobacteria and lactate were higher in the gut.
• Fecal butyrate and propionate levels were inversely associated with cognitive scores.
• SIgA levels were correlated with Proteobacteria abundance and lactate metabolite.

## 1 Introduction

Neurodevelopmental disorders are a group of disorders including intellectual disabilities, autistic spectrum disorders (ASD), attention-deficit/hyperactivity disorder (ADHD), communication disorders and learning disabilities, that manifest early in childhood and result in lifelong deficits in cognitive, social, emotional, academic, and adaptive functioning (Ismail and Shapiro, 2019; Thapar et al., 2017). The prevalence rates of children affected by the most common neurodevelopmental disorders are 0.7–2.2% for ASD, 7.9–9.5% for ADHD, 1.2–24% for dyslexia, and 1.4–19% for motor coordination disorder, as reviewed by Francés et al. (2022). Environmental factors are the primary drivers of neurodevelopment in very early childhood (Tucker-Drob and Briley, 2014) and epigenetic variations in brain signaling pathways can be induced by early life environmental exposures (Miguel et al., 2019). The pathophysiology of neurodevelopmental disorders is poorly understood, and consequently, treatment options are limited. However, growing evidence suggests that the development of these disorders may involve the gut microbiota and the immune system. If confirmed, these findings could open new opportunities for interventions (Fung et al., 2017).

Previous epidemiological case-control studies have shown that the gut microbiota is altered in children with neurodevelopmental disorders such as ASD (Dan et al., 2020; Finegold et al., 2010; Hsiao et al., 2013; Tomova et al., 2015; Wang et al., 2013) or ADHD (Jiang et al., 2018; Richarte et al., 2021; Szopinska-Tokov et al., 2020; Wang et al., 2022). A few prospective population-based studies have also identified differences in gut microbiota composition in children with lower scores on neurodevelopmental scales, compared to those with higher scores, and most emphasize the importance of genera from the phylum Bacteroidetes (Aatsinki et al., 2019; Carlson et al., 2018; Loughman et al., 2020; Sordillo et al., 2019; Tamana et al., 2021). While it is becoming increasingly clear that gut microbiota influences brain function and behavior through signaling pathways of the microbiota-gut-brain axis (Cryan et al., 2019; Nagpal and Cryan, 2021), clarity of the mechanisms through which these systems communicate is lacking.

Immunoglobulin A (IgA) is an antibody synthesized in the bone marrow, spleen, and lymph nodes by IgA-producing B cells and is also secreted into the gut as secretory IgA (SIgA). After birth, the gut mucosal immune system relies on SIgA provided by breast milk to supplement the newborn's initially low production (Battersby and Gibbons, 2013; Brandtzaeg, 2013; Kawano and Emori, 2013; Maruyama et al., 2009). SIgA binds pathogenic microbes and prevents their penetration of gut mucosa, representing a first-line immune defense against microbial infection (Mantis et al., 2011; Peterson et al., 2007; Yang and Palm, 2020). SIgA binds to commensal bacteria as well, which is critical for the development of gut microbiota during infancy by preventing overgrowth of a single species (Corthésy, 2013). It appears that gut microbiota can self-regulate the production and the microbial binding characteristics of SIgA via the short-chain fatty acid (SCFA) metabolites they produce (Kim et al., 2016). In IgA-producing B cells, it was demonstrated that SCFAs increase acetyl-CoA and regulate metabolic sensors to increase oxidative phosphorylation, glycolysis and fatty acid synthesis, which produce energy and building blocks supporting IgA production (Kim et al., 2016). Thus, a balanced gut microbiota during infancy promotes beneficial SIgA responses (Honda and Littman, 2016; Lin and Zhang, 2017). As an example, Yang et al. demonstrated that human-derived isolates of specific strains of *Bacteroides ovatus* induce high production of SIgA in the large intestines of germ-free mice (Yang et al., 2020).

Serum IgA deficiency has been noted in studies of children with ASD (Spiroski et al., 2009; Warren et al., 1997; Wasilewska et al., 2012). On the other hand, Zhou et al. found higher SIgA concentrations in stool samples from autistic patients than those from children whose neurodevelopmental trajectories were unremarkable (Zhou et al., 2018). Furthermore, these small-scale studies did not relate their results to the gut microbiota composition of the autistic children. To our knowledge, there is no epidemiological study on how the composition of gut microbiota during infancy may impact neurodevelopment specifically in regard to SIgA levels.

Using data from the large population-based CHILD (Canadian Healthy Infant Longitudinal Development) Cohort Study, we investigated: 1) the associations between gut microbiota (3–4 and 12 months), bacterial metabolites (3–4 months), and Bayley cognitive scores at age 12 and 24 months; 2) whether SIgA levels (3–4 months) mediate the association between infant gut microbiota and bacterial metabolites in early (3–4 months) infancy and neurodevelopmental delay at ages 12 and 24 months; and 3) whether infant gut microbiota in infancy (12 months) mediate the association between SIgA levels (3–4 months) and neurodevelopmental delay in children at 12 and 24 months.

## 2 Material and methods

### 2.1 Study design and population

This study of 178 full-term infants is a subset of the CHILD Cohort Study with complete neurodevelopment, gut microbiota and SIgA level assessments. Mothers of infant study subjects were enrolled during their second or third trimester of pregnancy from Edmonton study site between January 2009 and December 2012. Mothers were retained in the cohort if their newborns were a singleton live birth at ≥35 weeks of gestation with a birth weight greater than 2.5 kg. In vitro fertilized births and home births were excluded. This study was approved by the Human Research Ethics Board at the University of Alberta.

### 2.2 Data assessment

#### 2.2.1 Gut microbiota and gut bacterial metabolites

Infant fecal samples were collected at 3–4 (mean age, 3.7 months) and 12 months (mean age, 12.0 months) during planned study visits. Within the funding scope of the CHILD Cohort Study, this collection point strategically represented a sample during the peak of breastfeeding in Canadian infants. Stool samples were utilized to evaluate gut microbiota composition by sequencing the V4 region of the 16S ribosomal RNA (rRNA) gene using paired-end sequencing of PCR amplicons on the Illumina MiSeq platform and by processing using QIIME (v1.6.0) (Bolyen et al., 2019). PCR reactions for each sample were performed in triplicate with a negative control in each run with the following conditions: an initial denaturation step for 3 min at 94 °C, 20 cycles of denaturation for 30 s at 94 °C, annealing for 30 s at 50 °C and an extension step for 30 s at 7 °C. Taxonomic assignment at 97% sequence similarity against the GREENGENES database (version 13.8) was achieved to determine the relative abundance (OTUs, Operational Taxon Units) of microbial taxa. The fecal burden of *Clostrioides difficile* was quantified by qPCR (Drall et al., 2019). To identify microbiota enterotypes according to phylum-level relative abundance, samples were clustered using the partitioning around medoids (PAM) clustering algorithm (Arumugam et al., 2011), and the optimal number of clusters was determined according to the Calinski-Harabasz index and Silhouette width. Details about sample collection, DNA extraction and amplification (Azad et al., 2015, 2016) and clustering (Tamana et al., 2021) methods have been previously described. Finally, gut bacterial metabolite concentrations were quantified in micromoles per gram of feces (μmole/g) were assessed by Nuclear Magnetic Resonance (NMR) spectroscopy (Bridgman et al., 2017) in the 3–4 months fecal samples.

#### 2.2.2 SIgA levels

SIgA at 3–4 months level was quantified in milligrams per gram of wet-weight feces (mg/g) by SIgA enzyme-linked immunosorbent assay (Kang et al., 2018). A threshold of 5 mg/g feces was arbitrarily chosen to delimit children with a high SIgA level from those with a low SIgA level in our study, as no clear physiological SIgA values have been defined in infants (Granger et al., 2022), since these levels vary considerably depending on the infant's breastfeeding status (Battersby and Gibbons, 2013; Brandtzaeg, 2013; Kawano and Emori, 2013; Maruyama et al., 2009).

#### 2.2.3 Neurodevelopment

Infant neurodevelopmental assessments, using the Bayley Scale of Infant Development Third Edition (BSID-III), was completed at 12 and 24 months of age, during the day at a time when parents felt their infant was most alert (i.e., not during a scheduled naptime). The BSID-III is a validated objective measure of cognitive, language, and motor development for infants aged 1–42 months. In our study, we focused on the BSID-III cognitive scale (91-items) which assesses visual preference, attention, memory, exploration, manipulation, and concept formation (Bayley, 2005). A registered psychologist trained research staff to administer the BSID-III instrument and conducted semi-annual quality review of adherence to standardization procedures. Testing of participants was completed during a single session by one research staff. All scores were obtained based on the child's chronological age at the time of testing. Raw scores were converted to scaled scores, then to composite scores. The standardized population mean for the composite score is 100 (standard deviation of 15). A higher score on the BSID-III scales indicates greater abilities.

#### 2.2.4 Covariates

Hospital birth records provided data on infant sex and delivery mode. Information on maternal characteristics, such as ethnicity (categorical variable based on self-identified Caucasian versus other), age (numeric variable from birth date), education level (numeric variable as years of education), and prenatal smoking (categorical variable from positive response to smoking during pregnancy), as well as the infant characteristics, including gestational age (numeric variable) and breastfeeding duration at 3–4 months (categorical variable based on age formula-feeding started) were reported by standardized questionnaires completed by parents. Potential covariates included breastfeeding status (Lamberti et al., 2011; Rogier et al., 2014), birth mode (Chen et al., 2021; Rutayisire et al., 2016), gestational age and birth weight (Kumbhare et al., 2019), maternal ethnicity (Gupta et al., 2017), family income and maternal education (Levin et al., 2016), maternal BMI before pregnancy (Singh et al., 2017), child sex (Martin et al., 2016), and smoking status during pregnancy (Xie et al., 2021). Directed Acyclic Graphs (DAG) were generated with the daggity.net program to identify putative confounding factors of the relationship between gut microbiota, SIgA, and Bayley cognitive scores without introducing collider bias (Shrier and Platt, 2008). By applying the change-in-estimate procedure in linear regression to the minimum set of confounding variables from the DAG model (Evans et al., 2012), several variable were identified as potential confounders requiring adjustment, specifically: child sex, gestational age, birth mode and breastfeeding status at 3–4 months (Figs. S1 and S2). A complete dataset characterizing SIgA at 3–4 months, gut microbiota parameters at 3–4 months, and Bayley cognitive scores at 12 or 24 months, was available for mediation analysis for 178 and 163 infants, respectively. A complete dataset characterizing SIgA at 3–4 months, gut microbiota parameters at 12 months and Bayley cognitive scores at 12 or 24 months was available for mediation analysis for 170 and 154 infants, respectively.

### 2.3 Statistical analysis

First, we conducted linear regressions 1) between gut microbiota taxa and bacterial metabolites at 3–4 months and Bayley cognitive scores at 12 and 24 months, adjusted on child sex, gestational age, birth mode and breastfeeding status at 3–4 months 2) between SIgA and Bayley cognitive scores at 12 and 24 months, and 3) between gut microbiota taxa and bacterial metabolites at 3–4 months and SIgA at the same age.

Second, we performed mediation analyses to determine whether SIgA level mediate the association between the most relevant taxa relative abundances and gut bacterial metabolites at 3–4 months and Bayley cognitive scores in 12- and 24-month-old children. A simple mediator path model was performed to determine the indirect effects: SIgA level at 3–4 months (M) acted as the mediator in the path between gut microbiota enterotypes, taxonomic abundances, and gut bacterial metabolites at 3–4 months (X) and Bayley cognitive scores at 12 and 24 months (Y). Gut microbiota enterotype at 3–4 months was a 3-category variable: Proteobacteria-dominant cluster (reference category), Firmicutes-dominant cluster and Bacteroidetes-dominant cluster. Depending on the data distribution, relative abundance of phylum Bacteroidetes, and the genera *Bacteroides, Lactobacillus*, and *Enterococcus* at 3–4 months were categorical variables (tertiles) while other gut microbiota variables at 3–4 months are Napierian logarithm transformed.

SIgA levels at 3–4 months = intercept_1_ + a(gut microbiota at 3–4 months) + e_1_

Bayley cognitive score = intercept_2_ + c’(gut microbiota at 3–4 months) + b(SIgA levels at 3–4 months) + e_2_

Bayley cognitive score = intercept_3_ + c(gut microbiota at 3–4 months) + e_3_

Third, we performed mediation analyses to determine whether the gut microbiota at 12 months mediated the association between the SIgA level at 3–4 months and Bayley cognitive scores in 12- and 24-month-old children. A simple mediator pathway model was assessed to determine the indirect effects: gut microbiota at 12 months (M) acted as the mediator between SIgA level at 3–4 months (X) and Bayley cognitive scores at 12 and 24 months (Y). Gut microbiota enterotype at 12 months was a 3-category variable: Firmicutes-dominant cluster (reference category), *Bacteroides*-Firmicutes-dominant cluster and Bacteroidetes-dominant cluster. Depending on the data distribution, relative abundance of phylum Bacteroidetes, and the genera *Lactobacillus* and *Enterococcus* at 12 months were categorical variables (tertiles), relative abundance of the variable genus *Bacteroides* was not transformed and other gut microbiota variables at 12 months were Napierian logarithm transformed variables.

gut microbiota at 12 months = intercept_1_ + a(SIgA levels at 3–4 months) + e_1_

Bayley cognitive score = intercept_2_ + c’(SIgA levels at 3–4 months) + b(gut microbiota at 12 months) + e_2_

Bayley cognitive score = intercept_3_ + c(SIgA levels at 3–4 months) + e_3_

For both models, we used the diagonally weighted least squares (DWLS) estimator, specifically designed for ordinal data, and the procedure of bootstrapping (with 5000 replicates) was used to generate 95% confidence intervals for the mediation analysis. Low SIgA levels at 3–4 months (<5 mg/g feces) were used as the reference group. The composite scores of Bayley cognitive scales were analyzed as continuous variables. Mediation analyses were not adjusted for covariates. Associations with p-value <0.05 were considered statistically significant. All statistical analyses were conducted using R (version 4.2.2) within RStudio (version 2022.12.0 + 353). Mediation analyses were performed using the Lavaan package in R (version 0.6–15) (Rosseel, 2012).

## 3 Results

### 3.1 Study population

Among the 178 mother-infant pairs included in our study, mothers had a median body mass index (BMI) before pregnancy of 25 kg/m^2^ (IQR: 22, 30) and a median age during pregnancy of 32 years (IQR: 29, 34). Forty-two percent of the mothers had a university degree, the majority were white (80%), married or common law (94%), and did not report smoking during pregnancy (94%). Family income in the past 12 months before inclusion was $80,000 or over for 71% of the participating families. Children were born at term at a median gestational age of 39 weeks (IQR: 38, 40) and mainly vaginally delivered (75%) with a median birth weight of 3.5 kg (IQR: 3.2, 3.8). Forty-four percent of the children were female, 31% of the children were not breastfed, while the rest of the children were exclusively (36%) or partially breastfed (34%) at 3–4 months of age (Table 1). Distribution of the most relevant taxonomic relative abundances at 4 and 12 months, bacterial metabolites at 3–4 months, SIgA level at 3–4 months and the Bayley cognitive score at 12 and 24 months of age among the children of CHILD Cohort Study are given Table S1. The median level (25th percentile-75th percentile) of SIgA at 3–4 months was 4.4 mg/g of feces (2.4–9). The median Bayley cognitive scores were 110 (100–115) at 12 months and 105 (95–115) at 24 months of age. The most abundant phyla in the children gut microbiota were Firmicutes with a median relative abundance of 26.4% (12.8–50.0) at 3–4 months and 42.4% (30.2–54.0) at 12 months, and Bacteroidetes with a median relative abundance of 29.8% (3.4–79.6) at 3–4 months and 44.5% (24.1–58.0) at 12 months. The most prominent genera at both 3–4 months and 12 months included *Bacteroides*, *Veillonella* and *Bifidobacterium*. Spearman correlations between gut microbiota most relevant variables at 4 and 12 months of age are given Fig. S3.

**Table 1:** Description of the participants characteristics.

Maternal and child characteristics	N^a^	Median (IQR); n (%)^a^
**Maternal age (years)**	178	32 (29, 34)
**Maternal BMI before pregnancy (kg/m**^**2**^**)**	168	24.7 (21.6, 29.9)
**Maternal marital status**	172	
Married or common law		162 (94%)
Divorced, separated or single		10 (5.8%)
**Maternal education**	172	
High school or less		13 (7.6%)
Some post secondary		72 (42%)
Postgrad degree		14 (8.1%)
University degree		73 (42%)
**Family income, in the past 12 months**	160	
<$40,000		6 (3.8%)
$40,000 - $79,999		40 (25%)
$80,000 and over		114 (71%)
**Maternal ethnicity**	178	
White		143 (80%)
Asian		16 (9.0%)
First nation		11 (6.2%)
Other		8 (4.5%)
**Smoking status during pregnancy**	172	
No		161 (94%)
Yes		11 (6.4%)
**Gestational age (weeks)**	178	39 (38, 40)
**Child sex**	178	
Female		78 (44%)
Male		100 (56%)
**Weight at birth (kg)**	178	3.5 (3.2, 3.8)
**Delivery mode**	178	
Vaginal		133 (75%)
C-section		45 (25%)
**Breastfeeding status at 3 months**	177	
None		54 (30.5%)
Partial		60 (33.9%)
Exclusive		63 (35.6%)

a Before imputation for missing data.

### 3.2 Direct relations between gut microbiota, SIgA and Bayley cognitive scores

First, we investigated direct relations between gut microbiota and bacterial metabolites at 3–4 months of age and the Bayley cognitive scores in multiple regression models adjusted for child sex, gestational age, delivery mode and breastfeeding status at 3–4 months, as suggested by our DAG (Figs. S1 and S2) and the change-in-estimate procedure. Higher relative abundance of Proteobacteria at 3–4 months of age was associated with higher Bayley scores at 12 and 24 months (p-values: 0.03 and 0.01, respectively). Higher abundance of an unnamed genus from Enterobacteriaceae family at 3–4 months of age was associated with higher Bayley cognitive score at 24 months (p-value: 0.005) while higher abundance of S*treptococcus* genus at 3–4 months of age was associated with higher Bayley cognitive score at 12 months (p-value: 0.01) and *Lactobacillus* genus at 3–4 months of age with lower Bayley cognitive score at 24 months (p-value: 0.04). Higher acetate and lactate metabolite levels at 3–4 months of age were associated with higher Bayley cognitive score at 24 months (p-values: 0.05 and 0.01, respectively) while higher propionate and butyrate metabolite levels at 3–4 months of age were associated with its decrease (p-values: 0.01 and 0.02, respectively). The significant associations between metabolite levels and the Bayley cognitive scores at 24 months are underscored by the coefficients of determination (R^2^), showing that 10–13% of the variance in the Bayley cognitive score are due to the metabolite levels. Results are shown in Table 2.

**Table 2:** Adjusted associations between gut microbiota at 3–4 months and Bayley cognitive scores at 12 and 24 months of age.

Gut microbiota parameters^a^	Bayley cognitive score at 12 months					Bayley cognitive score at 24 months				
	N^b^	Beta^c^	95% CI^d^	p-value	R squared	N^b^	Beta^c^	95% CI^d^	p-value	R squared
**Gut microbiota at 3**–**4 months**										
Gut microbiota enterotypes	177			0.61	0.04	158			0.41	0.07
Proteobacteria-dominant cluster	31 (18%)	–	–			28 (18%)	–	–		
Firmicutes-dominant cluster	57 (32%)	−2.22	−7.38, 2.94			51 (32%)	−1.88	−8.46, 4.70		
Bacteroidetes-dominant cluster	89 (50%)	−2.25	−6.99, 2.49			79 (50%)	−3.99	−10.08, 2.10		
Phylum Proteobacteria	177	1.65	0.16, 3.14	**0.03**	0.06	158	2.42	0.54, 4.31	**0.01**	0.09
Phylum Firmicutes	177	0.69	−1.19, 2.57	0.47	0.03	158	−0.90	−3.37, 1.57	0.47	0.06
Phylum Bacteroidetes	177			0.25	0.05	158			0.67	0.06
1st tertile		–	–				–	–		
2nd tertile		−1.99	−6.57, 2.59				0.42	−5.42, 6.26		
3rd tertile		−3.75	−8.21, 0.71				−1.86	−7.59, 3.87		
Phylum Actinobacteria	177	0.04	−0.85, 0.94	0.93	0.03	158	−0.46	−1.61, 0.69	0.43	0.06
Family Enterobacteriaceae (unnamed genus)	177	0.86	−0.40, 2.11	0.18	0.04	158	2.24	0.67, 3.81	**0.005**	0.10
Genus *Bacteroides*	177			0.30	0.04	158			0.74	0.06
1st tertile		–	–				–	–		
2nd tertile		−2.14	−6.51, 2.22				−2.02	−7.74, 3.70		
3rd tertile		−3.31	−7.69, 1.08				−1.57	−7.29, 4.15		
Genus *Bifidobacterium*	173	−0.17	−0.95, 0.60	0.66	0.03	154	−0.24	−1.25, 0.77	0.64	0.06
Genus *Veillonella*	175	−0.08	−0.91, 0.75	0.85	0.03	156	−0.26	−1.32, 0.80	0.63	0.06
Genus *Streptococcus*	177	1.47	0.35, 2.60	**0.01**	0.07	158	1.16	−0.29, 2.61	0.12	0.07
Genus *Lactobacillus*	177			0.72	0.03	158			**0.04**	0.08
No		–	–				–	–		
Yes		0.69	−3.12, 4.49				−5.10	−9.87, −0.33		
Genus *Enterococcus*	177			0.23	0.04	158			0.86	0.05
No		–	–				–	–		
Yes		2.22	−1.42, 5.87				−0.42	−5.09, 4.26		
*Clostridioides difficile*	159	−0.35	−0.99, 0.28	0.27	0.05	143	−0.11	−0.98, 0.75	0.79	0.05

**Gut microbiota at 12 months**										
Gut microbiota enterotypes	151			0.90	0.04	151			**0.05**	0.12
Firmicutes-dominant cluster	30 (20%)	–	–			30 (20%)	–	–		
Bacteroidetes-Firmicutes-dominant cluster	71 (47%)	1.01	−3.62, 5.64			71 (47%)	7.22	1.14, 13.31		
Bacteroidetes-dominant cluster	50 (33%)	0.45	−4.44, 5.34			50 (33%)	6.80	0.37, 13.23		
Phylum Proteobacteria	168	−1.23	−2.84, 0.38	0.13	0.06	152	−0.03	−2.29, 2.24	0.98	0.08
Phylum Firmicutes	168	−1.42	−4.95, 2.12	0.43	0.05	152	−3.16	−8.21, 1.89	0.22	0.09
Phylum Bacteroidetes	168			0.78	0.05	152			0.89	0.09
1st tertile		–	–				–	–		
2nd tertile		−0.51	−4.40, 3.38				0.79	−4.69, 6.27		
3rd tertile		1.01	−3.55, 5.56				1.56	−4.92, 8.04		
Phylum Actinobacteria	168	−0.34	−1.37, 0.69	0.51	0.05	152	−1.26	−2.64, 0.13	0.07	0.10
Genus *Bacteroides*	168	0.01	−0.06, 0.08	0.73	0.04	152	0.06	−0.03, 0.16	0.20	0.10
Family Lachnospiraceae (unnamed genus)	167	0.45	−1.42, 2.31	0.64	0.05	151	−1.51	−4.13, 1.11	0.26	0.09
Genus *Bifidobacterium*	162	−0.28	−1.28, 0.72	0.58	0.05	146	−0.78	−2.13, 0.56	0.25	0.10
Genus *Veillonella*	168	−0.82	−1.57, −0.07	**0.03**	0.07	152	−0.51	−1.61, 0.59	0.36	0.09
Genus *Faecalibacterium*	143	0.13	−0.61, 0.86	0.74	0.05	129	0.84	−0.14, 1.82	0.09	0.13
Genus *Streptococcus*	167	−1.25	−2.39, −0.12	**0.03**	0.06	151	−1.10	−2.74, 0.53	0.18	0.09
Genus *Lactobacillus*	168			0.13	0.06	152			0.36	0.09
No		–	–				–	–		
Yes		−2.64	−6.04, 0.77				2.21	−2.56, 6.98		
Genus *Enterococcus*	168			0.74	0.04	152			0.92	0.08
No		–	–				–	–		
Yes		−0.59	−4.08, 2.91				0.26	−4.63, 5.14		
*Clostridioides difficile*	127	0.48	−0.11, 1.08	0.11	0.07	114	−0.39	−1.18, 0.40	0.33	0.10

**Gut microbiota metabolites at 3**–**4 months**										
Acetate	128	−1.05	−4.70, 2.59	0.57	0.03	113	4.42	−0.03, 8.87	**0.05**	0.09
Propionate	128	−0.68	−2.78, 1.42	0.52	0.03	113	−3.12	−5.59, −0.66	**0.01**	0.11
Butyrate	128	−1.14	−2.81, 0.53	0.18	0.04	113	−2.32	−4.27, −0.38	**0.02**	0.10
Tryptophan	124	−0.75	−4.51, 3.00	0.69	0.03	109	−2.57	−7.10, 1.97	0.26	0.07
Lactate	117	−0.12	−1.62, 1.38	0.87	0.02	103	2.30	0.46, 4.13	**0.01**	0.13
Formate	107	2.36	−0.07, 4.78	0.06	0.07	92	1.42	−1.69, 4.54	0.37	0.10
Tyrosine	106	−1.20	−3.55, 1.14	0.31	0.05	91	0.05	−2.88, 2.98	0.97	0.09
Valerate	128	−0.25	−1.25, 0.74	0.61	0.03	113	0.25	−0.99, 1.49	0.69	0.06

a Gut microbiota enterotype at 3–4 months was a 3-category variable: Proteobacteria-dominant cluster (reference category), Firmicutes-dominant cluster, and Bacteroidetes-dominant cluster. Gut microbiota enterotype at 12 months was a 3-category variable: Firmicutes-dominant cluster (reference category), Bacteroidetes-Firmicutes dominant cluster and Bacteroidetes-dominant cluster. Relative abundance of phylum Bacteroidetes, and the genera *Bacteroides, Lactobacillus* and *Enterococcus* at 3–4 months and 12 months were categorical variables (tertiles, or detected *vs*. not detected). Other gut microbiota variables are Napierian logarithm transformed.

b Number of observations.

c Average change in Bayley cognitive score at 12 or 24 months when the gut microbiota parameter was multiplied by Euler number *e*, or compared to the reference category; adjusted on child sex, gestational age, delivery mode, and breastfeeding status at 3–4 months.

d CI: Confidence interval.

**Fig. 1:**
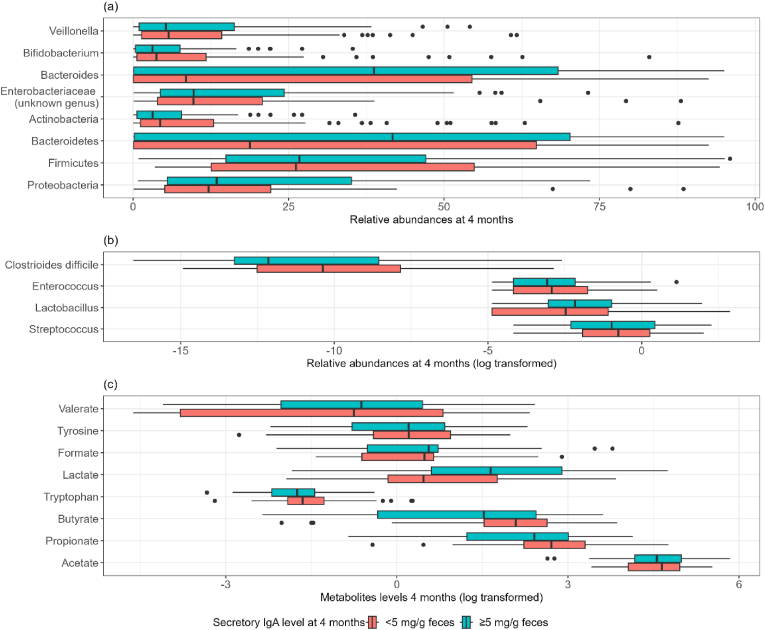
Distribution of gut bacteria abundances and metabolites at 3–4 months of age, depending on the level of SIgA at 3–4 months of age (n between 178 and 95). (a) Relative abundances of the most relevant gut microbiota taxa depending on secretory IgA level at 3–4 months of age (mg/g feces). (b) Napierian log transformed relative abundances of the most relevant gut microbiota taxa depending on SIgA level at 3–4 months of age (mg/g feces). (c)Napierian log transformed concentrations of the most relevant gut bacterial metabolites (μmole/g feces) depending on SIgA level at 3–4 months of age (mg/g feces).

Descriptively, distributions of the most relevant gut bacteria and their metabolites at 3–4 months of age depending on the level of SIgA at 3–4 months of age are given Fig. 1. Relative abundances of phylum Bacteroidetes, phylum Proteobacteria and genus *Bacteroides* at 3–4 months of age were higher in infants with high level of SIgA at 3–4 months of age, whereas relative abundance of *Clostridioides difficile* at 3–4 months of age was lower in infants with high levels of SIgA at 3–4 months of age. Valerate, formate and lactate metabolite concentrations at 3–4 months of age were higher in infants with high level of SIgA at 3–4 months, whereas tryptophan, butyrate, propionate and acetate metabolite concentrations at 3–4 months of age were lower in infants with high level of SIgA at 3–4 months of age (Fig. 1). Statistically, higher abundance of phylum Proteobacteria and a higher level of lactate metabolite at 3–4 months were associated with higher level of SIgA at 3–4 months (p-values: 0.03 and 0.01, respectively) while higher levels of propionate and butyrate at 3–4 months of age were associated with a lower level of SIgA at the same age (p-values: 0.005 and 0.003, respectively; Table S2).

Distribution of gut bacteria abundances at 12 months of age, depending on the level of SIgA at 3–4 months of age is given Fig. S4. Descriptively, relative abundances of Firmicutes and Proteobacteria phyla, and *Faecalibacterium*, *Streptococcus*, *Lactobacillus* and *Enterococcus* genera at 12 months of age were higher in infants with a high level of SIgA at 3–4 months of age (≥5 mg/g), whereas the relative abundance of Bacteroidetes and Actinobacteria phyla, *Bacteroides* genus, Lachnospiraceae (unknown genus) family and *Clostridioides difficile* at 12 months of age were lower in infants with a high level of SIgA at 3–4 months of age (Fig. S4).

SIgA level at 3–4 months tended to be associated with higher Bayley cognitive score at 24 months (p-value<0.10), but not at 12 months (Table S3).

Lastly, we reported the associations (multiple linear regression models) between the child characteristics (child sex, gestational age, delivery mode and breastfeeding status at 3–4 months) and the SIgA level at 3–4 months and the Bayley cognitive scores at 12 and 24 months. None of these child characteristics was associated with Bayley cognitive scores at 12 or 24 months, nor with SIgA levels, except for breastfeeding status at 3 months: exclusively and partially breastfed infants had higher SIgA levels at 3–4 months compared to non-breastfed infants (p < 0.001, Table S4).

### 3.3 Mediation analyses

#### 3.3.1 Relations between gut microbiota and bacterial metabolites at 3–4 months and Bayley cognitive scores in 12- and 24-month-old children, mediated by SIgA at 3–4 months

**Fig. 2:**
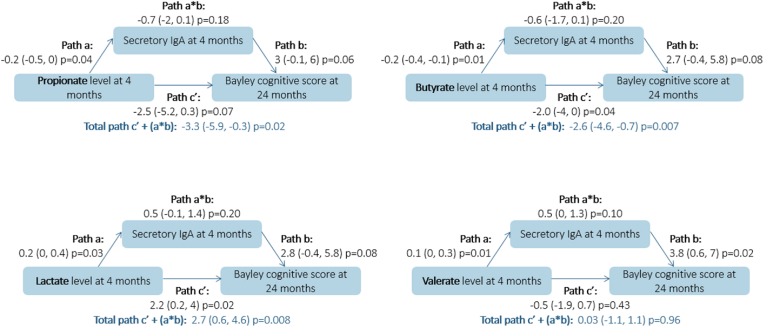
Associations between bacterial metabolites at 3–4 months and Bayley cognitive score at 24 months, mediated by SIgA levels at 3–4 months (n between 117 and 105). Bacterial metabolite variables (μmole/g feces) at 3–4 months are Napierian log-transformed. SIgA level at 3–4 months of age was a 2-category variable: low versus high level (cut-off 5 mg/g feces). Path a: direct relation between the predictor and the mediator. Path b: direct relation between the mediator and the outcome. Path c’: direct relation between the predictor and the outcome. Path ab: indirect relation between the predictor and the outcome. Path c' + (a∗b): total effect. Estimator was diagonally weighted least squares. Confidence Interval is at 95%.

Results of the relations between gut microbiota and bacterial metabolites at 3–4 months and Bayley cognitive scores in 12- and 24-month-old children, mediated by SIgA at 3–4 months are given Table 3 and Fig. 2. Relative abundance of *Bacteroides* genus and concentrations of lactate and valerate metabolites at 3–4 months were associated with a higher level of SIgA at 3–4 months whereas concentrations of propionate, butyrate and tryptophan at 3–4 months were associated with a lower level of SIgA at 3–4 months (p < 0.05, direct paths a). SIgA tended to be associated with increased Bayley cognitive score at 24 months of age (p < 0.10, direct path b). Propionate and butyrate levels at 3–4 months of age tended or were associated with decreased Bayley cognitive score at 24 months of age (direct paths c’: p = 0.07 and 0.04, respectively) but these associations were not statistically mediated by decreased SIgA level at 3–4 months of age (indirect paths a∗b, p = 0.18 and 0.2, respectively). Lactate level at 3–4 months of age was associated with increased Bayley cognitive score at 24 months of age (direct path c’: p = 0.02) but this association was not statistically mediated by increased SIgA level at 3–4 months of age (indirect path a∗b, p = 0.2). Finally, the level of SIgA at 3–4 months was not associated with Bayley cognitive score at 12 months in our study, and therefore did not statistically mediate any of the effects of gut microbiota or its metabolites at 3–4 months of age on Bayley cognitive score at 12 months.

**Table 3:** Relation between gut microbiota at 3–4 months and Bayley cognitive scores at 12 and 24 months of age, mediated by SIgA level at 3–4 months (sample size between 95 and 178).

Predictor^a^	Mediator^b^	Path^c^	Bayley cognitive score at 12 months				Bayley cognitive score at 24 months			
			N	Estimator^d^	CI95%^e^	p-value	N	Estimator^d^	CI95%^e^	p-value
Enterotypes at 3–4 months	SIgA at 3–4 months		178				163			
		a		0.19	−0.1, 0.5	0.16		0.21	0.0, 0.5	0.13
		b		−0.06	−2.2, 2.1	0.96		2.45	−0.3, 5.1	0.08
		c'		−1.04	−3.3, 1.3	0.37		−1.25	−4.2, 1.7	0.41
		ab		−0.01	−0.6, 0.5	0.97		0.51	−0.2, 1.6	0.26
		total: c' + (a∗b)		−1.05	−3.2, 1.1	0.33		−0.73	−3.8, 2.2	0.63
Proteobacteria at 3–4 months	SIgA at 3–4 months		178				163			
		a		0.11	−0.1, 0.3	0.20		0.08	−0.1, 0.3	0.35
		b		−0.42	−2.5, 1.7	0.70		2.12	−0.6, 4.7	0.12
		c'		1.66	−0.2, 3.4	0.07		1.98	0.3, 4.1	**0.04**
		ab		−0.05	−0.4, 0.3	0.76		0.18	−0.2, 0.8	0.48
		total: c' + (a∗b)		1.62	−0.2, 3.4	0.07		2.16	0.4, 4.3	**0.03**
Firmicutes at 3–4 months	SIgA at 3–4 months		178				163			
		a		−0.08	−0.3, 0.1	0.46		−0.10	−0.3, 0.1	0.36
		b		−0.14	−2.2, 2.0	0.90		2.23	−0.5, 4.9	0.11
		c'		0.53	−1.2, 2.3	0.55		−1.08	−3.0, 1.0	0.28
		ab		0.01	−0.3, 0.3	0.94		−0.22	−1.0, 0.3	0.49
		total: c' + (a∗b)		0.54	−1.1, 2.3	0.53		−1.30	−3.2, 0.7	0.19
Bacteroidetes at 3–4 months	SIgA at 3–4 months		178				163			
		a		0.11	−0.1, 0.3	0.36		0.11	−0.1, 0.4	0.37
		b		−0.08	−2.2, 2.0	0.94		2.32	−0.4, 5.0	0.10
		c'		−1.34	−3.3, 0.7	0.17		−0.17	−2.7, 2.3	0.89
		ab		−0.01	−0.4, 0.4	0.96		0.25	−0.3, 1.1	0.48
		total: c' + (a∗b)		−1.35	−3.2, 0.6	0.16		0.08	−2.3, 2.5	0.95
Actinobacteria at 3–4 months	SIgA at 3–4 months		178				163			
		a		−0.07	−0.2, 0.0	0.14		−0.08	−0.2, 0.0	0.15
		b		−0.16	−2.2, 2.0	0.89		2.28	−0.5, 5.1	0.11
		c'		0.05	−0.9, 1.1	0.92		−0.02	−1.2, 1.2	0.97
		ab		0.01	−0.2, 0.2	0.91		−0.18	−0.6, 0.1	0.33
		total: c' + (a∗b)		0.06	−0.9, 1.1	0.90		−0.20	−1.2, 0.8	0.71
Enterobacteriaceae (unnamed genus) at 3–4 months	SIgA at 3–4 months		178				163			
		a		0.04	−0.1, 0.2	0.56		0.02	−0.1, 0.2	0.80
		b		−0.24	−2.3, 1.9	0.82		2.27	−0.4, 4.9	0.09
		c'		0.87	−0.5, 2.2	0.22		1.86	0.4, 3.4	**0.02**
		ab		−0.01	−0.2, 0.2	0.91		0.04	−0.3, 0.5	0.82
		total: c' + (a∗b)		0.86	−0.5, 2.2	0.22		1.90	0.4, 3.5	**0.02**
*Bacteroides* at 3–4 months	SIgA at 3–4 months		178				163			
		a		0.21	0.0, 0.4	0.08		0.25	0.0, 0.5	**0.05**
		b		0.03	−2.0, 2.2	0.98		2.31	−0.4, 5.1	0.10
		c'		−1.52	−3.5, 0.6	0.15		−0.36	−3.1, 2.3	0.80
		ab		0.01	−0.6, 0.5	0.98		0.57	−0.1, 1.8	0.24
		total: c' + (a∗b)		−1.52	−3.5, 0.5	0.13		0.21	−2.2, 2.7	0.87
*Bifidobacterium* at 3–4 months	SIgA at 3–4 months		178				163			
		a		−0.03	−0.1, 0.1	0.44		−0.04	−0.1, 0.1	0.36
		b		0.11	−2.0, 2.2	0.92		2.52	−0.4, 5.3	0.08
		c'		−0.19	−1.1, 0.8	0.69		0.08	−1.0, 1.1	0.89
		ab		0.00	−0.1, 0.1	0.95		−0.11	−0.4, 0.1	0.46
		total: c' + (a∗b)		−0.19	−1.0, 0.7	0.68		−0.03	−1.0, 0.9	0.95
*Veillonella* at 3–4 months	SIgA at 3–4 months		178				163			
		a		−0.01	−0.1, 0.1	0.82		−0.01	−0.1, 0.1	0.81
		b		−0.14	−2.3, 2.0	0.90		2.34	−0.5, 5.1	0.10
		c'		−0.06	−1.0, 0.9	0.90		−0.08	−1.2, 1.0	0.88
		ab		0.00	−0.1, 0.1	0.98		−0.03	−0.3, 0.3	0.84
		total: c' + (a∗b)		−0.06	−1.0, 0.9	0.90		−0.11	−1.1, 0.9	0.84
*Streptococcus* at 3–4 months	SIgA at 3–4 months		178				163			
		a		−0.01	−0.1, 0.1	0.86		−0.02	−0.2, 0.1	0.81
		b		−0.13	−2.2, 1.9	0.90		2.37	−0.3, 5.0	0.09
		c'		1.46	0.3, 2.6	**0.01**		0.93	−0.5, 2.4	0.21
		ab		0.00	−0.1, 0.2	0.98		−0.04	−0.4, 0.3	0.83
		total: c' + (a∗b)		1.46	0.3, 2.6	**0.01**		0.89	−0.5, 2.4	0.23
*Lactobacillus* at 3–4 months	SIgA at 3–4 months		178				163			
		a		−0.06	−0.5, 0.3	0.76		−0.06	−0.5, 0.4	0.80
		b		−0.17	−2.2, 1.9	0.88		2.29	−0.4, 5.0	0.10
		c'		0.08	−3.4, 3.5	0.96		−3.57	−8.4, 1.2	0.15
		ab		0.01	−0.5, 0.6	0.96		−0.13	−1.4, 1.0	0.83
		total: c' + (a∗b)		0.09	−3.3, 3.5	0.96		−3.70	−8.4, 1.0	0.12
Enterococcus at 3–4 months	SIgA at 3–4 months		178				163			
		a		−0.38	−0.8, 0.0	0.06		−0.24	−0.7, 0.2	0.26
		b		0.05	−2.1, 2.2	0.96		2.29	−0.5, 5.0	0.11
		c'		2.67	−1.0, 6.4	0.16		−0.33	−4.6, 4.2	0.89
		ab		−0.02	−1.1, 1.0	0.97		−0.56	−2.2, 0.5	0.41
		total: c' + (a∗b)		2.65	−0.9, 6.2	0.14		−0.89	−5.0, 3.5	0.68
*Clostridioides difficile* at 3–4 months	SIgA at 3–4 months		160				148			
		a		−0.06	−0.1, 0.0	0.09		−0.05	−0.1, 0.0	0.19
		b		−0.64	−2.9, 1.4	0.56		1.94	−0.9, 4.7	0.18
		c'		−0.21	−0.9, 0.4	0.52		−0.19	−0.9, 0.6	0.61
		ab		0.04	−0.1, 0.2	0.63		−0.10	−0.4, 0.1	0.45
		total: c' + (a∗b)		−0.17	−0.8, 0.5	0.60		−0.29	−1.0, 0.4	0.43
Acetate metabolite at 3–4 months	SIgA at 3–4 months		129				117			
		a		−0.04	−0.4, 0.4	0.85		0.14	−0.2, 0.5	0.51
		b		−0.83	−3.6, 1.9	0.61		3.57	0.5, 14.1	0.24
		c'		−0.86	−4.5, 2.6	0.64		2.52	−3.5, 6.3	0.28
		ab		0.03	−0.6, 0.7	0.96		0.49	−0.9, 7.2	0.79
		total: c' + (a∗b)		−0.83	−4.3, 2.5	0.63		3.01	−0.6, 6.9	0.12
Propionate metabolite at 3–4 months	SIgA at 3–4 months		129				117			
		a		−0.28	−0.5, −0.1	**0.009**		−0.24	−0.5, 0.0	**0.04**
		b		−1.10	−3.8, 1.6	0.43		3.00	−0.1, 6.0	0.06
		c'		−0.86	−3.0, 1.3	0.43		−2.54	−5.2, 0.3	0.07
		ab		0.31	−0.5, 1.3	0.48		−0.72	−2.0, 0.1	0.18
		total: c' + (a∗b)		−0.55	−2.6, 1.4	0.60		−3.26	−5.9, −0.3	**0.02**
Butyrate metabolite at 3–4 months	SIgA at 3–4 months		129				117			
		a		−0.27	−0.4, −0.1	**0.002**		−0.22	−0.4, −0.1	**0.01**
		b		−1.33	−4.1, 1.4	0.34		2.71	−0.4, 5.8	0.08
		c'		−1.08	−2.8, 0.6	0.22		−2.04	−4.0, 0.0	**0.04**
		ab		0.35	−0.4, 1.2	0.38		−0.59	−1.7, 0.1	0.20
		total: c' + (a∗b)		−0.73	−2.3, 0.8	0.35		−2.63	−4.6, −0.7	**0.007**
Tryptophan metabolite at 3–4 months	SIgA at 3–4 months		125				113			
		a		−0.41	−0.8, 0.0	**0.05**		−0.43	−0.9, 0.0	**0.05**
		b		−0.96	−3.7, 1.6	0.48		3.64	0.5, 6.9	**0.04**
		c'		−0.89	−4, 2.4	0.59		−1.30	−5.0, 2.6	0.53
		ab		0.39	−0.8, 1.9	0.55		−1.56	−4.4, 0.0	0.28
		total: c' + (a∗b)		−0.50	−3.7, 2.8	0.76		−2.86	−6.5, 0.5	0.11
Lactate metabolite at 3–4 months	SIgA at 3–4 months		118				105			
		a		0.22	0.1, 0.4	**0.006**		0.19	0.0, 0.4	**0.03**
		b		−0.37	−3.1, 2.3	0.79		2.80	−0.4, 5.8	0.08
		c'		−0.04	−1.5, 1.5	0.96		2.19	0.2, 4.0	**0.02**
		ab		−0.08	−0.8, 0.6	0.80		0.52	−0.1, 1.4	0.20
		total: c' + (a∗b)		−0.12	−1.6, 1.2	0.86		2.71	0.6, 4.6	**0.008**
Formate metabolite at 3–4 months	SIgA at 3–4 months		108				96			
		a		0.14	−0.1, 0.4	0.28		0.08	−0.2, 0.4	0.60
		b		−1.57	−4.8, 1.4	0.31		4.38	0.8, 8.1	**0.02**
		c'		2.48	−0.2, 5.3	0.08		1.79	−2.0, 5.0	0.32
		ab		−0.22	−1.2, 0.4	0.56		0.34	−1.1, 1.8	0.64
		total: c' + (a∗b)		2.25	−0.4, 4.8	0.09		2.13	−1.7, 5.1	0.22
Tyrosine metabolite at 3–4 months	SIgA at 3–4 months		107				95			
		a		−0.06	−0.3, 0.2	0.61		0.01	−0.2, 0.3	0.92
		b		−1.31	−4.5, 1.7	0.41		4.53	0.8, 8.2	**0.02**
		c'		−1.35	−3.6, 0.8	0.24		−0.48	−3.1, 2.1	0.71
		ab		0.08	−0.4, 0.8	0.77		0.06	−1.3, 1.5	0.93
		total: c' + (a∗b)		−1.27	−3.5, 0.9	0.27		−0.42	−2.9, 2.0	0.74
Valerate metabolite at 3–4 months	SIgA at 3–4 months		129				117			
		a		0.07	0.0, 0.2	0.17		0.14	0.0, 0.3	**0.01**
		b		−0.75	−3.5, 1.9	0.59		3.83	0.6, 7.0	**0.02**
		c'		−0.19	−1.2, 0.8	0.71		−0.52	−1.9, 0.7	0.43
		ab		−0.05	−0.4, 0.2	0.68		0.54	0.0, 1.3	0.10
		total: c' + (a∗b)		−0.24	−1.2, 0.8	0.62		0.03	−1.1, 1.1	0.96

a Gut microbiota enterotype at 3–4 months was a 3-categoy variable: Proteobacteria-dominant cluster (reference category), Firmicutes-dominant cluster and Bacteroidetes-dominant cluster. Relative abundance of phylum Bacteroidetes, and genera *Bacteroides*, *Lactobacillus* and *Enterococcus* at 3–4 months are categorical variables (tertiles, or detected *vs*. not detected). Other gut microbiota variables are Napierian logarithm transformed.

b SIgA level at 3–4 months of age was a 2-category variable: low versus high level (cut off 5 mg/g).

c a: direct relation between the predictor and the mediator; b: direct relation between the mediator and the outcome; c: direct relation between the predictor and the outcome; ab: indirect relation between the predictor and the outcome; c' + (a∗b): total effect.

d Estimator: diagonally weighted least squares.

e CI95%: Confidence Interval at 95%.

#### 3.3.2 Relations between SIgA at 3–4 months and Bayley cognitive scores in 12- and 24-month-old children, mediated by gut microbiota at 12 months

Results of the relations between SIgA level and Bayley cognitive scores in 12- and 24-month-old children, mediated by gut microbiota at 12 months are given Table 4. Higher level of SIgA at 3–4 months was associated with increased relative abundance of Proteobacteria phylum (direct path a, p-value: 0.01). Higher abundance of Proteobacteria phylum was associated with decreased Bayley cognitive score at 12 months (direct path b, p-value: 0.04), but not at 24 months, whereas higher relative abundance of *Bacteroides* genus at 12 months was associated with an increased Bayley cognitive score at 24 months of age (direct path b, p-value: 0.04). Gut microbiota dominated by Bacteroidetes phylum at 12 months of age (enterotypes) was also associated with an increased Bayley cognitive score at 24 months, compared to children with gut microbiota dominated by Firmicutes phylum (direct path b, p-value: 0.04). Finally, relation between SIgA at 3–4 months and Bayley cognitive scores at 12 and 24 months of age were not statistically mediated by the gut microbiota at 12 months of age.

**Table 4:** Relation between SIgA level at 3–4 months and Bayley cognitive scores at 12 and 24 months of age, mediated by gut microbiota at 12 months (sample size between 129 and 170).

Predictor^a^	Mediator^b^	Path^c^		Bayley cognitive score at 12 months			Bayley cognitive score at 24 months			
			N	Estimator^d^	CI95%^e^	p-value	N	Estimator^d^	CI95%^e^	p-value
SIgA at 3–4 months	Enterotypes at 12 months		152				153			
		a		−0.17	−0.5, 0.2	0.36		−0.14	−0.5, 0.2	0.44
		b		0.07	−2.0, 2.3	0.95		2.54	0.1, 5.0	**0.04**
		c'		−0.05	−3.5, 3.3	0.98		3.23	−1.4, 7.7	0.17
		ab		−0.01	−0.7, 0.5	0.97		−0.36	−1.6, 0.6	0.51
		total: c' + (a∗b)		−0.06	−3.5, 3.2	0.97		2.87	−1.7, 7.4	0.21
SIgA at 3–4 months	Proteobacteria at 12 months		170				154			
		a		0.43	0.1, 0.7	**0.006**		0.40	0.1, 0.7	**0.01**
		b		−1.51	−3.0, −0.1	**0.04**		−0.42	−2.4, 1.6	0.69
		c'		−0.03	−3.1, 3.1	0.99		3.05	−1.7, 7.7	0.19
		ab		−0.64	−1.6, 0.0	0.12		−0.17	−1.0, 0.8	0.71
		total: c' + (a∗b)		−0.68	−3.8, 2.5	0.68		2.88	−1.8, 7.5	0.21
SIgA at 3–4 months	Firmicutes at 12 months		170				154			
		a		0.06	−0.1, 0.2	0.42		−0.01	−0.2, 0.1	0.87
		b		−1.75	−5.2, 1.8	0.33		−4.40	−9.0, 0.2	0.06
		c'		−0.57	−3.7, 2.6	0.73		2.83	−1.8, 7.4	0.22
		ab		−0.10	−0.6, 0.3	0.60		0.06	−0.7, 0.9	0.88
		total: c' + (a∗b)		−0.68	−3.8, 2.5	0.68		2.88	−1.8, 7.5	0.21
SIgA at 3–4 months	Bacteroidetes at 12 months		170				154			
		a		−0.10	−0.4, 0.2	0.58		0.06	−0.3, 0.4	0.73
		b		0.45	−1.3, 2.3	0.62		1.40	−0.8, 3.7	0.22
		c'		−0.63	−3.8, 2.6	0.70		2.80	−2.0, 7.4	0.23
		ab		−0.04	−0.6, 0.3	0.83		0.09	−0.6, 0.9	0.80
		total: c' + (a∗b)		−0.68	−3.8, 2.5	0.68		2.88	−1.8, 7.5	0.21
SIgA at 3–4 months	Actinobacteria at 12 months		170				154			
		a		−0.20	−0.7, 0.3	0.42		−0.20	−0.7, 0.3	0.46
		b		−0.33	−1.4, 0.6	0.52		−1.24	−2.5, 0.1	0.06
		c'		−0.74	−3.9, 2.5	0.66		2.64	−2.1, 7.1	0.25
		ab		0.07	−0.3, 0.4	0.71		0.25	−0.5, 1.1	0.53
		total: c' + (a∗b)		−0.68	−3.8, 2.5	0.68		2.88	−1.8, 7.5	0.21
SIgA at 3–4 months	*Clostridioides difficile* (num) at 12 months		129				116			
		a		−0.29	−1.3, 0.8	0.59		−0.57	−1.8, 0.7	0.36
		b		0.37	−0.3, 1.1	0.30		−0.25	−0.9, 0.3	0.40
		c'		−1.95	−5.4, 1.7	0.29		2.62	−2.3, 7.4	0.28
		ab		−0.11	−1.0, 0.3	0.74		0.15	−0.4, 0.8	0.60
		total: c' + (a∗b)		−2.06	−5.6, 1.6	0.26		2.77	−2.0, 7.5	0.25
SIgA at 3–4 months	*Bacteroides* at 12 months		170				154			
		a		−1.97	−9.5, 5.5	0.61		−1.11	−8.7, 6.6	0.78
		b		0.01	−0.1, 0.1	0.78		0.09	0.0, 0.2	**0.04**
		c'		−0.54	−3.9, 2.7	0.75		2.98	−1.7, 7.5	0.19
		ab		−0.02	−0.4, 0.3	0.90		−0.10	−1.0, 0.6	0.80
		total: c' + (a∗b)		−0.56	−3.9, 2.7	0.74		2.88	−1.8, 7.5	0.21
SIgA at 3–4 months	Lachnospiraceae (unnamed genus) at 12 months		170				154			
		a		−0.18	−0.5, 0.1	0.17		−0.23	−0.5, 0.0	0.10
		b		0.47	−1.1, 2.7	0.62		−1.68	−4.5, 0.9	0.21
		c'		−0.59	−3.8, 2.5	0.72		2.43	−2.2, 7.1	0.30
		ab		−0.09	−0.5, 0.3	0.64		0.39	−0.2, 1.2	0.29
		total: c' + (a∗b)		−0.68	−3.9, 2.5	0.68		2.82	−1.7, 7.3	0.22
SIgA at 3–4 months	*Bifidobacterium* at 12 months		170				154			
		a		0.27	−0.3, 0.8	0.32		0.28	−0.3, 0.9	0.36
		b		−0.31	−1.2, 0.6	0.52		−0.67	−2.0, 0.6	0.30
		c'		−0.27	−3.7, 3.0	0.88		3.12	−1.6, 8.0	0.19
		ab		−0.08	−0.6, 0.2	0.69		−0.18	−0.9, 0.4	0.56
		total: c' + (a∗b)		−0.35	−3.8, 2.9	0.84		2.93	−1.7, 7.8	0.22
SIgA at 3–4 months	*Veillonella* at 12 months		170				154			
		a		0.26	−0.4, 0.9	0.44		0.12	−0.6, 0.8	0.73
		b		−0.73	−1.5, 0.1	0.07		−0.71	−1.9, 0.5	0.24
		c'		−0.37	−3.7, 2.8	0.82		2.97	−1.7, 7.4	0.20
		ab		−0.19	−0.9, 0.3	0.52		−0.08	−0.8, 0.5	0.79
		total: c' + (a∗b)		−0.56	−3.9, 2.7	0.74		2.88	−1.8, 7.5	0.21
SIgA at 3–4 months	*Faecalibacterium* at 12 months		170				154			
		a		0.71	−0.1, 1.5	0.10		0.44	−0.4, 1.3	0.33
		b		0.03	−0.7, 0.8	0.93		0.85	−0.1, 1.8	0.08
		c'		−1.24	−4.9, 2.4	0.51		3.17	−1.8, 8.3	0.22
		ab		0.02	−0.6, 0.7	0.94		0.38	−0.4, 1.6	0.44
		total: c' + (a∗b)		−1.22	−4.8, 2.4	0.51		3.54	−1.4, 8.6	0.16
SIgA at 3–4 months	*Streptococcus* at 12 months		170				154			
		a		0.42	0.0, 0.9	0.06		0.33	−0.1, 0.8	0.16
		b		−1.10	−2.3, 0.0	0.06		−1.20	−2.9, 0.3	0.15
		c'		−0.44	−3.6, 2.7	0.79		3.45	−1.0, 7.8	0.13
		ab		−0.47	−1.4, 0.1	0.22		−0.39	−1.3, 0.3	0.34
		total: c' + (a∗b)		−0.91	−4.1, 2.3	0.58		3.05	−1.5, 7.5	0.19
SIgA at 3–4 months	*Lactobacillus* at 12 months		170				154			
		a		0.33	0.0, 0.8	0.11		0.20	−0.2, 0.6	0.34
		b		−1.74	−3.8, 0.3	0.10		0.85	−2.1, 3.7	0.56
		c'		0.02	−3.3, 3.2	0.99		2.71	−2.0, 7.3	0.25
		ab		−0.58	−1.9, 0.2	0.32		0.17	−0.6, 1.4	0.72
		total: c' + (a∗b)		−0.56	−3.9, 2.7	0.74		2.88	−1.8, 7.5	0.21
SIgA at 3–4 months	*Enterococcus* at 12 months		170				154			
		a		0.16	−0.2, 0.6	0.45		0.13	−0.3, 0.6	0.54
		b		−0.33	−2.5, 1.8	0.76		0.45	−2.6, 3.4	0.77
		c'		−0.51	−3.8, 2.7	0.76		2.82	−1.9, 7.5	0.23
		ab		−0.05	−0.8, 0.5	0.86		0.06	−0.8, 0.9	0.88
		total: c' + (a∗b)		−0.56	−3.9, 2.7	0.74		2.88	−1.8, 7.5	0.21

a SIgA was a 2-category variable: low versus high level (cut off 5 mg/g).

b Gut microbiota enterotype at 12 months was a 3-category variable: Firmicutes-dominant cluster (reference category), Bacteroidetes-Firmicutes-dominant cluster and Bacteroidetes-dominant cluster. Relative abundance of phylum Bacteroidetes phylum, and genera *Bacteroides*, *Lactobacillus* and *Enterococcus* at 12 months are categorical variables (tertiles, or detected vs. not detected). Other gut microbiota variables are Napierian logarithm transformed.

c a: direct relation between the predictor and the mediator; b: direct relation between the mediator and the outcome; c: direct relation between the predictor and the outcome; ab: indirect relation between the predictor and the outcome; c' + (a∗b): total effect.

d Estimator: diagonally weighted least squares.

e CI95%: Confidence Interval at 95%.

## 4 Discussion

### 4.1 Overview

In this birth cohort study of 178 infants, we investigated relationships between the early-life gut microbiome and immune system with future neurodevelopment. Extending our previous 12-month gut microbiota findings (Tamana et al., 2021), we found higher cognitive scores at 24 months of age among infants with higher fecal lactate levels at 3–4 months (beta: 2.3, 95%CI: 0.5–4.1, p < 0.01), with higher abundance of Proteobacteria at 3–4 months (beta: 2.4, 95%CI: 0.5–4.3, p = 0.01) and its family Enterobacteriaceae at 3–4 months (beta: 2.2, 95%CI: 0.7–3.8, p < 0.01) but with lower butyrate (beta: 2.3, 95%CI: 4.3–0.4, p = 0.02) and propionate (beta: 3.1, 95%CI: 5.6 to −0.7, p = 0.01) levels at 3–4 months. Cross-sectionally at 3–4 months, SIgA levels were positively correlated with Proteobacteria abundance and lactate levels, and negatively related to butyrate and propionate levels. SIgA levels at 3–4 months tended to be associated with higher 24-month cognitive scores (p < 0.1). The mediation analyses by SIgA of the relationship between gut bacterial metabolites and Bayley cognitive scores did not reach statistical significance for all associations.

### 4.2 Comparison with the literature and biological plausibility

Our findings support the hypothesis that early-life gut bacterial metabolite production may play an important role for cognitive development. This accords with the growing body of literature on the microbiota-gut-brain axis positing that the gut microbiota play a crucial role in brain development and function (Cryan et al., 2019). SCFAs such as butyrate and propionate are a major class of signaling molecules produced by bacterial fermentation of dietary carbohydrates, odd-chain fatty acids and some proteins (Al-Lahham et al., 2010; Zoetendal and de Vos, 2014). They directly affect the host digestive tract through energy metabolism enhancement, anti-inflammatory effects and maintenance of the intestinal barrier, but the role of SCFAs in neurodevelopment is controversial. Lobzhanidze et al. found that an intraperitoneal injection of propionic acid (175 mg/kg) in adolescent rats induced brain alteration by significantly decreasing the total number of synaptic vesicles, presynaptic mitochondria and synapses with a symmetric active zone (Lobzhanidze et al., 2020). In other animal studies, the treatment of rodents with SCFAs (predominantly propionate) was found to induce behavioral changes (Lobzhanidze et al., 2020; Shams et al., 2019; Shultz et al., 2008) similar to those of our epidemiological study, while another study found that butyrate increased sociability and downregulated the activity-related transcriptome in the frontal cortex in an autism mouse model (Kratsman et al., 2016). Children with ASD have been reported to have both lower (Adams et al., 2011) and higher (Wang et al., 2012) fecal SCFAs levels than controls. These findings highlight the challenges of assigning unambiguous potential roles for SCFAs in neurodevelopment and indicate the need for more research. In our study, the associations between propionate and butyrate levels with both lower SIgA levels and decreased Bayley-III cognitive score suggest that while these two SCFAs may generally be beneficial for gut health, their early-life balance may have nuanced implications for neurodevelopment. This aligns with emerging research indicating that while SCFAs support brain health through various mechanisms, excessive levels early in life (as shown with direct administration of SCFAs in rodent model experiments) could disrupt gut-brain signaling and immune responses, leading to altered neurodevelopment (Dalile et al., 2019).

Lactate, another metabolite produced by gut bacteria such as *Lactobacillus* and *Bifidobacterium* spp., plays an important role in promoting the proliferation of intestinal stem cells, thus facilitating intestinal epithelial development (Lee et al., 2018). It is also an important substrate in the gut that can be utilized and converted into SCFAs, such as butyrate and propionate (Duncan et al., 2004). Consistently, lactate levels in our study were negatively correlated with butyrate and propionate levels. The positive impact of lactate levels on Bayley cognitive score at 24 months suggests that lactate may support cognitive development. Lactate is readily taken up by the infant brain facilitating myelin regeneration, and is a signaling molecule for processes such as memory and neuroprotection (Deng et al., 2023).

As expected, SIgA levels at 3–4 months were associated with the breastfeeding status at 3 months, consistent with evidence that the infant gut mucosal immune system relies on SIgA provided by breast milk to supplement the initially low production (Battersby and Gibbons, 2013; Brandtzaeg, 2013; Kawano and Emori, 2013; Maruyama et al., 2009). SIgA plays a crucial role intestinal health, by protecting mucosal surfaces against pathogens and regulating interactions with commensal microbes. We previously identified Proteobacteria and lactate metabolite as candidate pathways in the infant gut that stimulate SIgA levels (Chen et al., 2023). By preserving mucosal integrity and homeostasis (Corthésy, 2013), SIgA could influence neurodevelopmental outcomes through effects on the gut-brain axis. In this study, the trend of an association between SIgA levels at 3–4 months and Bayley cognitive score at 24 months (beta: 1.9, 95%CI: 0.3-4.1, p = 0.08) favors this hypothesis. However, the mediation analyses by SIgA of the relationship between gut bacterial metabolites and Bayley cognitive scores did not reach statistical significance for all associations. Furthermore, the absence of a mediating effect of SIgA on cognitive score at 12 months suggests that the timing and developmental stages might influence how gut microbiota and SIgA interact to affect neurodevelopment. Larger studies are needed to investigate these mechanisms.

### 4.3 Strengths and limits

Our study was based on a subsample of the prospective Canadian CHILD Cohort Study including 178 mother-child pairs with child gut microbiota data, SIgA level assessment, Bayley cognitive scores evaluated at two different stages of development and rich questionnaire data to characterize the population. High throughput deep sequencing permitted to profile gut microbiota at a critical period of microbiota and experts have conducted objective assessment of neurodevelopment using a well validated and widely used gold standard measure. This is the first study to evaluate the putative causal relationships of gut microbiota to SIgA levels on neurological development as measured by Bayley cognitive scores at 12 and 24 months. Cross-sectional associations between gut microbiota and SIgA level at 3–4 months of age should be interpreted with caution, as SIgA levels may influence gut microbiota and lead to a risk of reverse causality. Future studies should also consider a shotgun metagenomic approach to examine microbial function with greater depth to further assess causality and mechanisms. We were also unable to examine infants at high risk for neurodevelopmental morbidity because the CHILD Cohort Study excluded preterm birth below 35 weeks. Other groups insufficiently represented in our study included families of low socioeconomic status and non-white ethnicity. Further studies are required to investigate the generalizability of our findings to other populations.

### 4.4 Conclusion

This study underscores the complexity of the interactions between gut microbiota, bacterial metabolites, and SIgA levels in early life, and their collective impacts on early neurodevelopment. It contributes to the emerging understanding of how the gut microbiota and immune system interact in infancy, and how in turn, these interactions may influence neurodevelopment. The potential mediating role of SIgA offers a novel perspective on the mechanisms involved, highlighting the importance of the microbiota-gut-immune-brain axis in early development. Given the critical nature of early childhood for brain development, a better understanding of these relations could provide valuable insights into preventing or mitigating cognitive development issues.

## CRediT authorship contribution statement

**Aline Davias:** Writing – review & editing, Writing – original draft, Visualization, Resources, Methodology, Investigation, Formal analysis, Conceptualization. **Myah Verghese:** Writing – review & editing, Writing – original draft. **Sarah L. Bridgman:** Writing – review & editing, Project administration, Data curation. **Hein M. Tun:** Writing – review & editing, Data curation. **Catherine J. Field:** Writing – review & editing, Funding acquisition, Data curation. **Matthew Hicks:** Writing – review & editing, Funding acquisition. **Jacqueline Pei:** Writing – review & editing, Resources, Data curation. **Anne Hicks:** Writing – review & editing, Funding acquisition. **Theo J. Moraes:** Writing – review & editing, Funding acquisition. **Elinor Simons:** Writing – review & editing, Funding acquisition. **Stuart E. Turvey:** Writing – review & editing, Funding acquisition. **Padmaja Subbarao:** Writing – review & editing, Funding acquisition. **James A. Scott:** Writing – review & editing, Funding acquisition, Data curation. **Piushkumar J. Mandhane:** Writing – review & editing, Resources, Funding acquisition, Data curation. **Anita L. Kozyrskyj:** Writing – review & editing, Writing – original draft, Validation, Supervision, Software, Resources, Project administration, Methodology, Investigation, Funding acquisition, Data curation, Conceptualization.

## Declaration of competing interest

The authors declare the following financial interests/personal relationships which may be considered as potential competing interests: Anita L. Kozyrskyj reports financial support was provided by 10.13039/501100000024Canadian Institutes of Health Research. Anita L. Kozyrskyj reports financial support was provided by Women and Children's 10.13039/100022895Health Research Institute. If there are other authors, they declare that they have no known competing financial interests or personal relationships that could have appeared to influence the work reported in this paper.

## Data Availability

Data will be made available on request.

## Acronyms

ADHD: attention-deficit/hyperactivity disorder
ASD: autistic spectrum disorders
BSID-III: Bayley scale of infant development, third edition
CHILD: Canadian healthy infant longitudinal development cohort study
DWLS: diagonally weighted least squares
DAG: directed acyclic graph
IgA: immunoglobulin A
NMR: nuclear magnetic resonance
PAM: partitioning around medoids
rRNA: ribosomal RNA
SCFA: short-chain fatty acid
sIgA: secretory IgA
