# Supplementary material for: Gut microbiota metabolites, secretory immunoglobulin A and Bayley-III cognitive scores in children from the CHILD Cohort Study
Source: Brain Behav Immun Health. 2025 Jan 15;44:100946. doi: 10.1016/j.bbih.2025.100946 (PMC11795817; doi:10.1016/j.bbih.2025.100946)
Supplement: Multimedia component 1 [file mmc1.docx]

Gut microbiota metabolites, secretory immunoglobulin A and Bayley-III cognitive scores in children from the CHILD Cohort Study

**Supplementary files**

Aline Davias^a,b^, Myah Verghese^a^, Sarah L. Bridgman^a^, Hein M. Tun^c,d^, Catherine J. Field^e^, Matthew Hicks^a^, Jacqueline Pei^f^, Anne Hicks^a^, Theo J. Moraes^g^, Elinor Simons^h^, Stuart E. Turvey^i^, Padmaja Subbarao^g,j^, James A. Scott^j^, Piushkumar J. Mandhane^a,k^, Anita L. Kozyrskyj^a^.

^1^Edmonton Clinic Health Academy, Department of Pediatrics, Faculty of Medicine and Dentistry, University of Alberta, Edmonton, Canada.

^2^Environmental Epidemiology applied to Development and Respiratory Health team, Institute for Advanced Biosciences, University Grenoble Alpes, Inserm, CNRS, 38700 La Tronche, France.

^3^The Jockey Club School of Public Health and Primary Care, Li Ka Shing Institute of Health Sciences, Faculty of Medicine, The Chinese University of Hong Kong, Hong Kong, SAR, China.

^4^Microbiota I-Center (MagIC), Hong Kong, SAR, China.

^5^Department of Agricultural, Food and Nutritional Science, Faculty of Agricultural, Life and Environmental Sciences, University of Alberta, Edmonton, Canada.

^6^Department of Educational Psychology, Faculty of Education, University of Alberta, Edmonton, Canada.

^7^Hospital for Sick Children (SickKids), Department of Pediatrics, University of Toronto, Toronto, Canada.

^8^Children’s Hospital Research Institute of Manitoba, Department of Pediatrics and Child Health, University of Manitoba, Winnipeg, Canada.

^9^BC Children's Hospital, Department of Pediatrics, Faculty of Medicine, University of British Columbia, Vancouver, Canada.

^10^Dalla Lana School of Public Health, Division of Occupational and Environmental Health, University of Toronto, Toronto, Canada.

^11^Faculty of Medicine and Health Sciences, UCSI University, Kuala Lumpur, Malaysia.

[Table S1: Distribution of the SIgA level at 3–4 months of age, the gut microbiota at 4 and 12 months of age and the Bayley cognitive scale at 12 and 24 months of age in the children from the Canadian CHILD Cohort Study. 3](#_Toc182402329)

[Table S2: Associations between gut microbiota taxa and bacterial metabolites at 3–4 months and SIgA at 3–4 months. 4](#_Toc182402330)

[Table S3: Associations between SIgA at 3–4 months and the Bayley cognitive scores at 12 and 24 months of age. 6](#_Toc182402331)

[Table S4: Effects of the child characteristics on the SIgA at 3–4 months and on the Bayley cognitive scores at 12 and 24 months of age. 7](#_Toc182402332)

[Figure S1: Directed Acyclic Graph of the relation between gut microbiota at 3–4 months and Bayley cognitive scores in infancy, mediated by child SIgA level at 3–4 months. 8](#_Toc182325061)

[Figure S2: Directed Acyclic Graph of the relation between SIgA level at 3–4 months and Bayley cognitive scores in infancy, mediated by child gut microbiota at 12 months. 9](#_Toc182325062)

[Figure S3: Spearman correlations between gut microbiota variables at 4 and 12 months of age. 10](#_Toc182325063)

[Figure S4: Distribution of gut bacteria abundances at 12 months of age, depending on the level of SIgA at 3–4 months of age (n=170). 11](#_Toc182325064)

Table S1: Distribution of the SIgA level at 3–4 months of age, the gut microbiota at 4 and 12 months of age and the Bayley cognitive scale at 12 and 24 months of age in the children from the Canadian CHILD Cohort Study.

| **Characteristics^1^** | **Min** | **Q1** | **Median** | **Mean** | **Q3** | **Max** | **N** |
| --- | --- | --- | --- | --- | --- | --- | --- |
| **SIgA at 3–4 months** | 0.6 | 2.4 | 4.4 | 6.4 | 9 | 40 | 178 |
| **Child cognitive composite score at 12 months** | 75 | 100 | 110 | 110 | 115 | 145 | 178 |
| **Child cognitive composite Score at 24 months** | 70 | 95 | 105 | 106 | 115 | 145 | 158 |
| **Child gut microbiota at 3–4 months** |  |  |  |  |  |  |  |
| Phylum Proteobacteria | 0.1 | 5.2 | 12.4 | 18.4 | 26.0 | 89.0 | 178 |
| Phylum Firmicutes | 0.9 | 12.8 | 26.4 | 34.2 | 50.0 | 96.0 | 178 |
| Phylum Bacteroidetes | 0.0 | 0.1 | 29.8 | 34.6 | 70.0 | 95.0 | 178 |
| Phylum Actinobacteria | 0.0 | 1.1 | 4.0 | 8.9 | 12.0 | 88.0 | 178 |
| Family *Enterobacteriaceae* (unnamed genus) | 0.1 | 3.9 | 9.7 | 15.7 | 21.0 | 88.0 | 178 |
| Genus Bacteroides | 0.0 | 0.1 | 22.3 | 30.4 | 61.0 | 95.0 | 178 |
| Genus *Bifidobacterium* | 0.0 | 0.5 | 3.2 | 7.8 | 11.0 | 83.0 | 178 |
| Genus *Veillonella* | 0.0 | 1.1 | 5.5 | 10.7 | 15.0 | 62.0 | 178 |
| Genus *Streptococcus* | 0.0 | 0.1 | 0.5 | 1.1 | 1.0 | 10.0 | 178 |
| Genus *Lactobacillus* | 0.0 | 0.0 | 0.0 | 0.2 | 0.0 | 18.0 | 178 |
| Genus *Enterococcus* | 0.0 | 0.0 | 0.0 | 0.1 | 0.0 | 3.0 | 178 |
| *Clostridioides difficile* | 0.0 | 0.0 | 0.0 | 0.0 | 0.0 | 0.0 | 160 |
| Acetate metabolite | 14.0 | 63.6 | 103.5 | 111.2 | 146.0 | 345.0 | 128 |
| Propionate metabolite | 0.4 | 7.3 | 14.2 | 19.4 | 25.0 | 117.0 | 128 |
| Butyrate metabolite | 0.1 | 2.7 | 7.2 | 9.4 | 13.0 | 48.0 | 128 |
| Tryptophan metabolite | 0.0 | 0.1 | 0.2 | 0.2 | 0.0 | 1.0 | 124 |
| Lactate metabolite | 0.1 | 1.0 | 2.7 | 11.3 | 11.0 | 116.0 | 117 |
| Formate metabolite | 0.1 | 0.6 | 1.7 | 2.6 | 2.0 | 44.0 | 107 |
| **Child gut microbiota at 12 months** |  |  |  |  |  |  |  |
| Phylum Proteobacteria | 0.2 | 1.9 | 3.8 | 6.0 | 7.0 | 53.0 | 124 |
| Phylum Firmicutes | 12.6 | 30.0 | 42.4 | 44.1 | 54.0 | 94.0 | 124 |
| Phylum Bacteroidetes | 0.0 | 24.1 | 44.5 | 40.4 | 58.0 | 85.0 | 124 |
| Phylum Actinobacteria | 0.0 | 0.9 | 2.5 | 5.4 | 6.0 | 43.0 | 124 |
| Genus *Bacteroides* | 0.0 | 11.2 | 33.0 | 33.8 | 54.0 | 85.0 | 124 |
| Family *Lachnospiraceae* (unnamed genus) | 0.3 | 4.0 | 7.5 | 8.6 | 11.0 | 42.0 | 124 |
| Genus *Bifidobacterium* | 0.0 | 0.7 | 2.1 | 4.8 | 5.0 | 42.0 | 124 |
| Genus *Veillonella* | 0.0 | 0.2 | 1.4 | 4.9 | 7.0 | 42.0 | 124 |
| Genus *Faecalibacterium* | 0.0 | 0.0 | 1.7 | 4.2 | 6.0 | 36.0 | 124 |
| Genus *Lactobacillus* | 0.0 | 0.0 | 0.0 | 0.0 | 0.0 | 1.0 | 124 |
| Genus *Enterococcus* | 0.0 | 0.0 | 0.0 | 0.0 | 0.0 | 1.0 | 124 |
| Genus *Streptococcus* | 0.0 | 0.1 | 0.3 | 0.8 | 1.0 | 7.0 | 124 |
| *Clostridioides difficile* | 0.0 | 0.0 | 0.0 | 0.1 | 0.0 | 3.0 | 94 |
| ^1^SIgA at 3–4 months are in mg/g feces. Gut microbiota taxa are relative abundances. Gut bacterial metabolites are in µmole/g feces. | | | | | | | |

Table S2: Associations between gut microbiota taxa and bacterial metabolites at 3–4 months and SIgA at 3–4 months.

| **Gut microbiota parameters^1^** | **SIgA at 3–4 months (mg/g feces)** | | | | |
| --- | --- | --- | --- | --- | --- |
|  | **N^2^** | **Beta^3^** | **95% CI^4^** | **p-value** | **R squared** |
| **Gut microbiota enterotypes at 3–4 months** | 178 |  |  | 0.37 | 0.01 |
| Proteobacteria-dominant cluster | 31 (17%) | — | — |  |  |
| Firmicutes-dominant cluster | 57 (32%) | -0.18 | -0.56, 0.21 | 0.36 |  |
| Bacteroidetes-dominant cluster | 90 (51%) | 0.02 | -0.34, 0.38 | 0.90 |  |
| **Phylum Proteobacteria at 3–4 months** | 178 | 0.13 | 0.01, 0.24 | **0.03** | 0.03 |
| **Phylum Firmicutes at 3–4 months** | 178 | -0.02 | -0.15, 0.12 | 0.80 | 0.00 |
| **Phylum Bacteroidetes at 3–4 months** | 178 |  |  | 0.30 | 0.01 |
| 1^st^ tertile |  | — | — |  |  |
| 2^nd^ tertile |  | 0.24 | -0.08, 0.57 | 0.14 |  |
| 3^rd^ tertile |  | 0.04 | -0.27, 0.36 | 0.78 |  |
| **Phylum Actinobacteria at 3–4 months** | 178 | -0.04 | -0.11, 0.02 | 0.19 | 0.01 |
| **Family *Enterobacteriaceae* (unnamed genus) at 3–4 months** | 178 | 0.06 | -0.03, 0.16 | 0.20 | 0.01 |
| **Genus *Bacteroides* at 3–4 months** | 178 |  |  | 0.67 | 0.01 |
| 1^st^ tertile |  | — | — |  |  |
| 2^nd^ tertile |  | 0.10 | -0.22, 0.43 | 0.53 |  |
| 3^rd^ tertile |  | 0.13 | -0.20, 0.46 | 0.44 |  |
| **Genus *Bifidobacterium* at 3–4 months** | 174 | -0.01 | -0.07, 0.05 | 0.82 | 0.00 |
| **Genus *Veillonella* at 3–4 months** | 176 | -0.02 | -0.08, 0.04 | 0.54 | 0.002 |
| **Genus *Streptococcus* at 3–4 months** | 178 | 0.01 | -0.07, 0.10 | 0.74 | 0.001 |
| **Genus *Lactobacillus* at 3–4 months** | 178 |  |  | 0.63 |  |
| No |  | — | — |  |  |
| Yes |  | 0.07 | -0.21, 0.35 | 0.63 | 0.001 |
| **Genus *Enterococcus* at 3–4 months** | 178 |  |  | 0.15 |  |
| No |  | — | — |  |  |
| Yes |  | -0.21 | -0.48, 0.07 | 0.15 | 0.01 |
| ***Clostridioides difficile* at 3–4 months** | 160 | -0.04 | -0.09, 0.00 | 0.07 | 0.02 |
| **Acetate metabolite at 3–4 months** | 129 | 0.05 | -0.21, 0.31 | 0.69 | 0.001 |
| **Propionate metabolite at 3–4 months** | 129 | -0.20 | -0.35, -0.06 | **0.005** | 0.06 |
| **Butyrate metabolite at 3–4 months** | 129 | -0.17 | -0.27, -0.06 | **0.003** | 0.07 |
| **Tryptophan metabolite at 3–4 months** | 125 | -0.09 | -0.36, 0.18 | 0.52 | 0.003 |
| **Lactate metabolite at 3–4 months** | 118 | 0.14 | 0.03, 0.24 | **0.01** | 0.05 |
| **Formate metabolite at 3–4 months** | 108 | 0.07 | -0.10, 0.24 | 0.42 | 0.006 |
| **Tyrosine metabolite at 3–4 months** | 107 | 0.01 | -0.16, 0.17 | 0.94 | 0.00 |
| **Valerate metabolite at 3–4 months** | 129 | 0.04 | -0.03, 0.12 | 0.27 | 0.01 |
| ^1^Gut microbiota enterotype at 3–4 months was a 3-category variable: Proteobacteria-dominant cluster (reference category), Firmicutes-dominant cluster and Bacteroidetes-dominant cluster. Relative abundance of phylum Bacteroidetes, and the genera *Bacteroides*, *Lactobacillus* and *Enterococcus* at 3–4 months were categorical variables (tertiles, or detected vs. not detected). Other gut microbiota variables are Napierian logarithm transformed.  ^2^Number of observations.  ^3^Average change in SIgA at 3–4 months when the gut microbiota parameter was multiplied by Euler number *e*, or compared to the reference category.  ^4^CI: Confidence Interval. | | | | | |

Table S3: Associations between SIgA at 3–4 months and the Bayley cognitive scores at 12 and 24 months of age.

|  | **Bayley cognitive score at 12 months** | | | | |  | **Bayley cognitive score at 24 months** | | | | |
| --- | --- | --- | --- | --- | --- | --- | --- | --- | --- | --- | --- |
|  | **N^1^** | **Beta^2^** | **95% CI^3^** | **p-value** | **R squared** |  | **N^1^** | **Beta^2^** | **95% CI^3^** | **p-value** | **R squared** |
| **SIgA at 3–4 months (mg/g feces)** | 241 | 0.09 | -1.43, 1.62 | 0.91 | 0.000 |  | 214 | 1.91 | -0.26, 4.07 | **0.08** | 0.014 |
| ^1^Number of observations. ^2^Average change in Bayley cognitive scores at 1 or 2 years when the SIgA at 3–4 months was multiplied by Euler number *e*. ^3^CI: Confidence Interval. | | | | | | | | | | | |

Table S4: Effects of the child characteristics on the SIgA at 3–4 months and on the Bayley cognitive scores at 12 and 24 months of age.

| **Child characteristics** | **SIgA at 3-4 months (mg/g feces)** | | | |  | **Bayley cognitive score at 12 months** | | | |  | **Bayley cognitive score at 24 months** | | | |
| --- | --- | --- | --- | --- | --- | --- | --- | --- | --- | --- | --- | --- | --- | --- |
|  | **N^1^** | **Beta^2^** | **95% CI^3^** | **p-value** |  | **N^1^** | **Beta^2^** | **95% CI^3^** | **p-value** |  | **N^1^** | **Beta^2^** | **95% CI^3^** | **p-value** |
| **Child sex** | 239 |  |  | 0.35 |  | 239 |  |  | 0.54 |  | 213 |  |  | 0.21 |
| Female |  | — | — |  |  |  | — | — |  |  |  | — | — |  |
| Male |  | -0.1 | -0.32, 0.11 |  |  |  | 0.9 | -1.97, 3.76 |  |  |  | -2.51 | -6.46, 1.45 |  |
| **Gestational age (weeks)** | 239 | -0.01 | -0.09, 0.06 | 0.72 |  | 239 | 0.68 | -0.29, 1.65 | 0.17 |  | 213 | 1.19 | -0.20, 2.59 | 0.09 |
| **Delivery mode** | 239 |  |  | 0.92 |  | 239 |  |  | 0.53 |  | 213 |  |  | 0.18 |
| Vaginal |  | — | — |  |  |  | — | — |  |  |  | — | — |  |
| C-section |  | 0.01 | -0.24, 0.27 |  |  |  | -1.06 | -4.39, 2.27 |  |  |  | -3.2 | -7.85, 1.46 |  |
| **Breastfeeding status at 3 months** | 239 |  |  | **<0.001** |  | 239 |  |  | 0.38 |  | 213 |  |  | 0.19 |
| None |  | — | — |  |  |  | — | — |  |  |  | — | — |  |
| Partial |  | 0.62 | 0.33, 0.91 |  |  |  | -2.62 | -6.42, 1.17 |  |  |  | 3.79 | -1.51, 9.10 |  |
| Exclusive |  | 0.98 | 0.70, 1.25 |  |  |  | -1.92 | -5.50, 1.66 |  |  |  | 4.51 | -0.50, 9.52 |  |
| ^1^Number of observations. ^2^Average changes in SIgA at 3-4 months, Bayley cognitive score at 12 months or 24 months depending on the child characteristics; adjusted for all the variables in the table.  ^3^CI: Confidence Interval. | | | | | | | | | | | | | | |

Figure S1: Directed Acyclic Graph of the relation between gut microbiota at 3–4 months and Bayley cognitive scores in infancy, mediated by child SIgA level at 3–4 months.


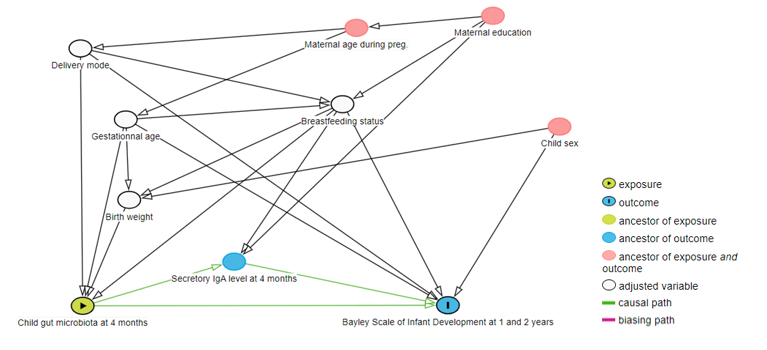


Associations were adjusted on all *a priori* identified covariates in white on the figure, so that no biasing path between the child gut microbiota at 4 months and the Bayley-III scale of Infant development at 12 and 24 months remains (which would appear in purple on the DAG).

Figure S2: Directed Acyclic Graph of the relation between SIgA level at 3–4 months and Bayley cognitive scores in infancy, mediated by child gut microbiota at 12 months.


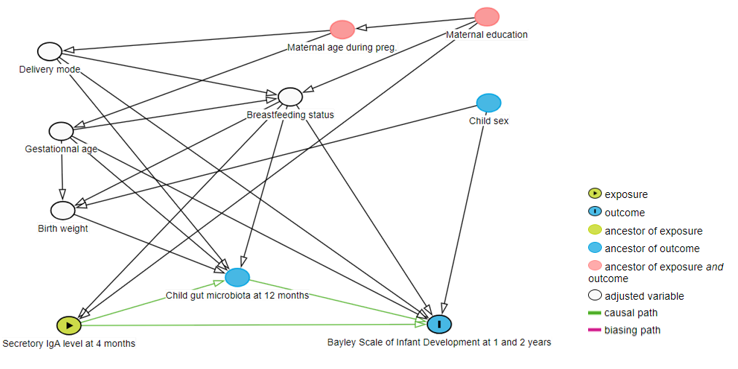


Associations were adjusted on all *a priori* identified covariates in white on the figure, so that no biasing path between the sIgA level at 4 months and the Bayley-III scale of Infant development at 12 and 24 months remains (which would appear in purple on the DAG).

Figure S3: Spearman correlations between gut microbiota variables at 4 and 12 months of age.


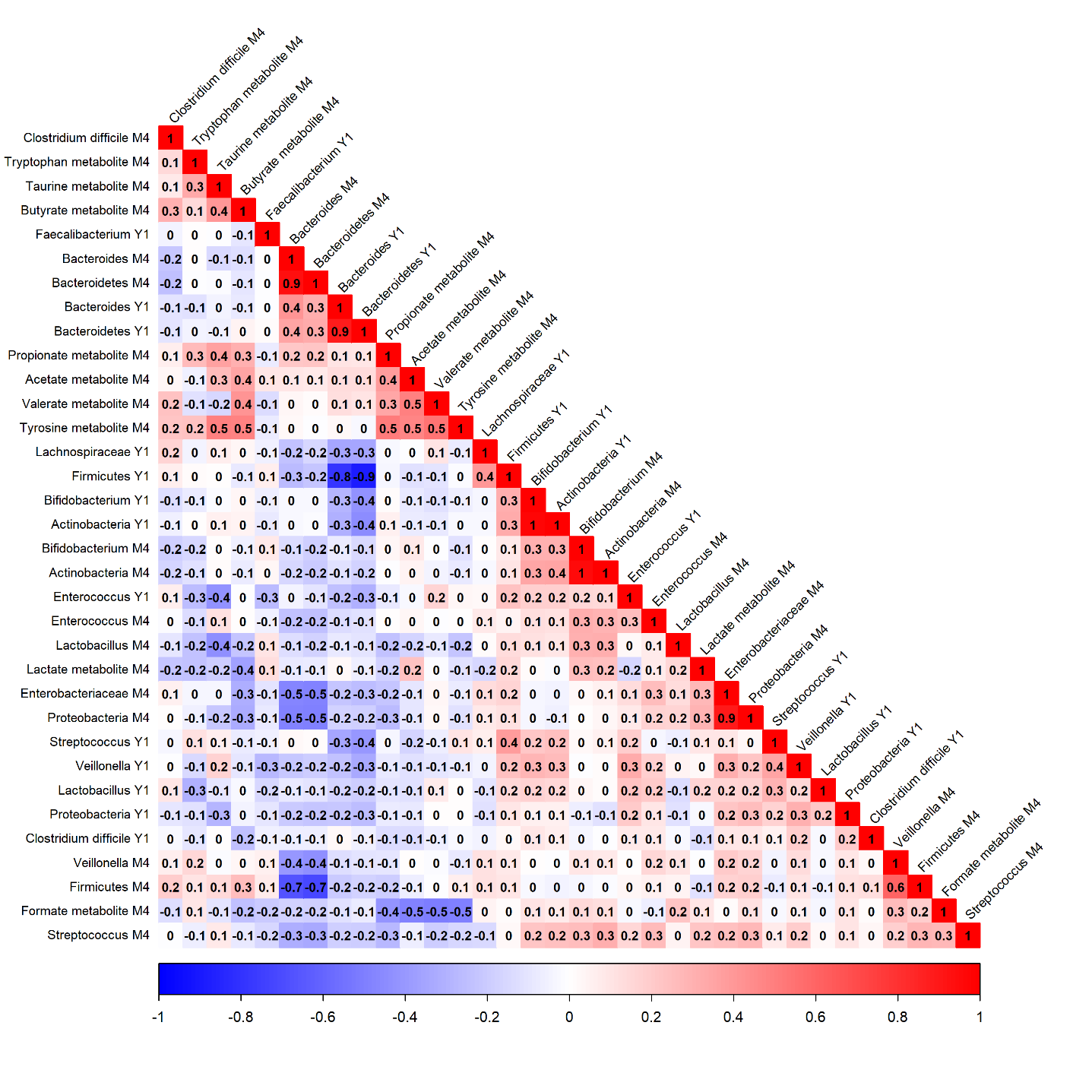


M4: assessed at 3–4 months, Y1: assessed at one year of age.

Figure S4: Distribution of gut bacteria abundances at 12 months of age, depending on the level of SIgA at 3–4 months of age (n=170).


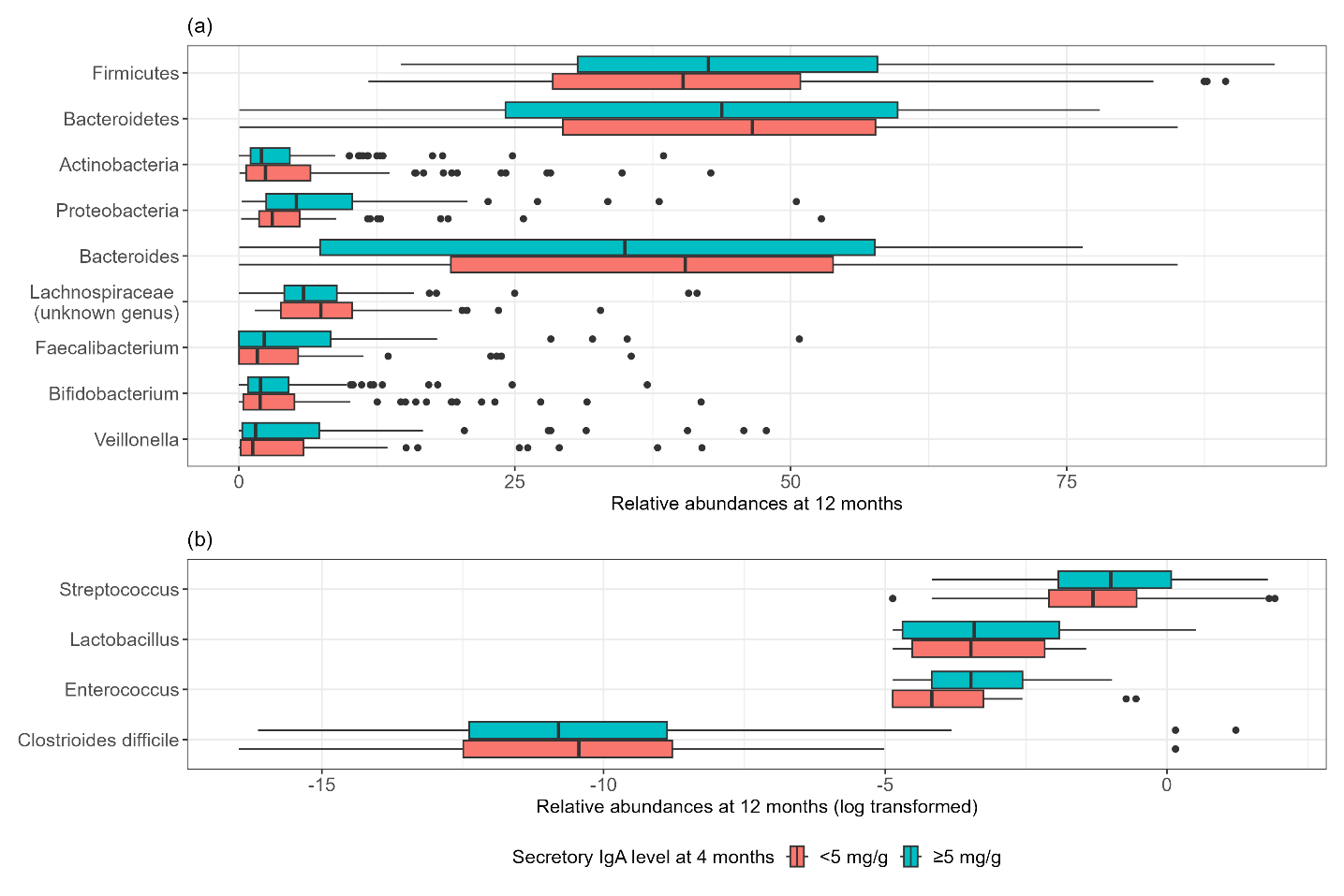


(a) Relative abundances of the most relevant gut microbiota taxa at 12 months depending on SIgA level at 3–4 months of age.

(b) Napierian log transformed relative abundances of the most relevant gut microbiota taxa at 12 months depending on SIgA level at 3–4 months of age.
